# Exploring the Prevalence and Factors Associated With Fatigue in Axial Spondyloarthritis in an Asian Cohort in Singapore

**DOI:** 10.3389/fmed.2021.603941

**Published:** 2021-02-04

**Authors:** Wei Ze Lim, Warren Fong, Yu Heng Kwan, Ying Ying Leung

**Affiliations:** ^1^Department of Rheumatology and Immunology, Singapore General Hospital, Singapore, Singapore; ^2^Yong Loo Lin School of Medicine, National University of Singapore, Singapore, Singapore; ^3^Duke-NUS Medical School, Singapore, Singapore

**Keywords:** fatigue, spondyloarthritis, disease activity, disease impact, ethnic

## Abstract

**Aim:** To evaluate the prevalence of fatigue and the factors associated with fatigue among patients with axial spondyloarthritis (axSpA) within an Asian population.

**Method:** We used the baseline data from a clinic registry in a tertiary referral center. All patients fulfilled the 2009 Assessment of SpondyloArthritis international Society (ASAS) classification criteria for axSpA. Severe fatigue was defined as Bath Ankylosing Spondylitis Disease Activity Index-fatigue (BASDAI-fatigue) ≥5/10 and vitality domain of Short Form-36 Health Survey (SF-36 VT) ≤10th percentile of the general population.

**Results:** We included 262 consecutive patients with axSpA (79% men, 82.4% Chinese). The mean (standard deviation, SD) age and duration of disease were 41.7 (13.7) and 10.1 (8.3) years, respectively. 145 (55.3%) and 52 (31.1%) patients reported severe fatigue by the BASDAI-fatigue and SF-36 VT criteria, respectively. Patients with severe fatigue had worse scores across all disease activity assessments and disease impact measures compared to those without severe fatigue. Using principal component analyses, disease activity and impact were associated with BASDAI-fatigue, while disease activity and impact, and disease chronicity were associated with SF-36 VT. In the univariable analyses, all disease activity assessments and disease impact measures correlated with both BASDAI-fatigue and SF-36 VT. In the multivariable analyses, BASDAI-axial pain, BASFI, BAS-G, and ethnicity were associated with BASDAI-fatigue, while ASQoL and BASDAI-morning stiffness were associated with SF-36 VT.

**Conclusion:** Fatigue is prevalent amongst patients with axSpA in Asia and is associated with disease activity, disease impact as well as patient related factors.

## Introduction

Axial spondyloarthritis (axSpA) is a chronic inflammatory rheumatic disease characterized by predominant involvement of the axial skeleton, with peak disease onset in late adolescence and early adulthood (1). Associated hallmark features of axSpA include enthesitis, peripheral arthritis, and extra-articular involvement such as anterior uveitis (2). Often striking patients in their prime of life, axSpA has wide-ranging and long-lasting impacts on different aspects of life, including functional, psychological and social domains (3).

Fatigue is widely regarded as a cardinal manifestation of axSpA and is one of the three most frequently reported symptoms alongside pain and stiffness (4), with a high prevalence ranging from 49 to 66.4% in different cohorts (5–10). The importance of fatigue has been recognized as evidenced by its inclusion in the Assessment of SpondyloArthritis international Society (ASAS) core set for clinical practice (11). Fatigue is generally described as a subjective sensation of tiredness that is relentless, pervasive, unpredictable, and unresolving (12, 13). It also has adverse impacts on how axSpA patients function in daily activities (7–9, 14–16), work performance (9, 16), and quality of life (6, 9, 14, 15). However, the mechanisms driving fatigue in axSpA remains unclear. Despite the advent of biologics, fatigue remains poorly treated in many (6), thus highlighting its complexity and the management challenge it poses. Current understanding holds that fatigue in spondyloarthritis is multi-factorial and associated with disease-specific parameters (5–10, 14, 15, 17), poor psychological health (7, 8, 10, 14, 15), sleep disturbance (10, 17), and women gender (5, 8), with disease activity being the single most important predictor.

Existing literature on fatigue in axSpA is largely derived from Western populations and data on Asians with axSpA is limited. The pathogenesis of axSpA is underpinned by genetic and environmental factors (18), and there is evidence that ethnicity and geographic differences (18–22) may shape the disease manifestations, severity and comorbidities in SpA. Moreover, it has been well-known that socio-cultural differences influence perceptions on health (23). Singapore is a multi-ethnic Asian country with a unique ethnic profile of Chinese, Malay, Indians, and others in Asia. In this study, we aim to evaluate the prevalence of fatigue and the factors associated with fatigue among patients with axSpA within an Asian population.

## Methods

### Study Population

We used the baseline data from the PRESPOND (PREcision medicine in SPONdyloarthritis for Better Outcomes and Disease Remission) registry (3, 24) in a tertiary referral center in Singapore. The PRESPOND registry recruited consecutive and all patients who attended a designated SpA clinic from 2012 to 2015 and fulfilled the 2009 ASAS classification criteria for axSpA (11). The study protocol was approved by the SingHealth Centralized Institutional Review Board (CIRB Ref: 2012/498/E) and written informed consent was obtained from each patient prior to their participation.

### Data Collection

Data was collected during clinic visit using a standardized protocol. Patients self-administered questionnaires in paper and pencil format, which captured socio-demographic information including age, gender, ethnicity, highest education level, housing type, and other patient reported outcomes (PROs). We collected data on highest education level and housing type on an ordinal scale of 1–7, adapted from the method used in the Singapore Census of Population 2010 (25, 26). Housing type has been used as a surrogate of socio-economic status in Singapore (27). Information on disease duration was retrieved from clinical records.

Patients were examined by designated rheumatologists (WF and YYL) for tender, swollen and damaged joints on a 66/68/68 diarthrodial joints diagram, as well as enthesitis using the Spondyloarthritis Research Consortium of Canada (SPARCC) enthesitis index (28). Spinal mobility parameters, including cervical rotation, tragus-to-wall distance, lateral lumbar flexion, lumbar flexion, and inter-malleolar distance, were measured twice and the better attempts were recorded. The Bath Ankylosing Spondylitis Metrology Index (BASMI_10_) score was then computed using the 10-step definition by Jones et al. (29). Body weight and height were measured in the clinic. Erythrocyte sedimentation rate (ESR) was measured as part of standard care.

### Assessment of Fatigue

Fatigue was assessed using two different measures—the Bath Ankylosing Spondylitis Disease Activity Index (BASDAI) fatigue item and the Short Form-36 Health Survey (SF-36) vitality (VT) domain.

The BASDAI-fatigue item is one of the five core symptoms evaluated in the BASDAI which has been validated for axSpA patients in Singapore (30). Patients rated the severity of their fatigue over the past week on a 0-10 numeric rating scale (NRS) (0: none, 10: very severe). We defined severe fatigue as BASDAI-fatigue ≥5/10 as with previous studies on fatigue and axSpA (5, 6, 9, 10, 31, 32). The SF-36 VT is one of the eight domains of the SF-36, a generic measure of health-related quality of life (HRQoL) validated for axSpA patients in Singapore (33). The SF-36 VT domain comprises of four items on the themes of fatigue and energy levels (feeling full of life, having lots of energy, feeling worn out, and feeling tired). Each item has five response options on a Likert scale and the total raw score is converted to a 0–100 point scale (0: lowest vitality, 100: highest vitality) (34). The SF-36 VT represents a reverse concept of fatigue (35), in which a lower score is indicative of greater fatigue. We defined severe fatigue as SF-36 VT ≤10th percentile of the general Singaporean population (8). The 10th percentile SF-36 VT score of the general population was calculated to be 47.6, by applying the formula (10th percentile = mean − 1.28 × standard deviation, SD) to the data obtained from the general Singaporean population (36).

### Other PROs

We collected data on BASDAI, including BASDAI-axial pain, BASDAI-peripheral joint pain, and BASDAI-morning stiffness (mean score of the questions on severity and duration), scored on a 0–10 NRS (0: none, 10: very severe). We assessed the effect of axSpA on patient's global well-being through the Bath Ankylosing Spondylitis Patient Global Score (BAS-G), which ranged from 0–100 (0: very good, 100: very bad) (37). Physical function was assessed through the Bath Ankylosing Spondylitis Functional Index (BASFI) (38). HRQoL was assessed using the SF-36 as well as the Ankylosing Spondylitis Quality of Life (ASQoL) (39), which has been validated in Singapore (40).

### Statistical Analysis

We described and compared the baseline characteristics between patients with and without severe fatigue. Differences in continuous variables were analyzed using the independent samples *t*-test or the Mann-Whitney *U* test as appropriate, while that of categorical variables were analyzed using the Chi-squared test. We conducted a principal component analysis (PCA) without rotation to evaluate for clusters of variables that represent clinically distinct concepts. We selected variables that were either theoretically linked to fatigue or described in literature to be associated with fatigue (5–10, 14, 15, 17, 19, 41). Only variables with pre-defined correlations of <0.6 were included in the model. The study rheumatologists discussed to reach a consensus on the inclusion or exclusion of variables with high collinearity (Spearman's rho > 0.6). Principal components with eigenvalues > 1 were entered into linear regression models for BASDAI-fatigue and SF-36 VT, respectively, to determine component(s) significantly associated with each measure of fatigue.

We performed univariable analysis using Spearman's rank correlation to determine the relationship between the study variables and measures of fatigue. In our study, we considered correlations of magnitude ≥ 0.5 as strong, 0.3–0.49 as moderate and 0.1–0.29 as weak (42). In the multivariable analysis, we evaluated for variables associated with each measure of fatigue using stepwise linear regression. Variables with *p* < 0.2 in the univariate analysis for association with fatigue were included. We also considered variables that either have a strong theoretical basis for association with fatigue or were found to be predictors of fatigue in previous studies.

All statistical analyses were carried out using IBM SPSS Statistic Package, version 25 (IBM, Armonk) and statistical significance was set at *p* < 0.05.

## Results

A total of 262 consecutive patients with axSpA were included in this study, of which 262 had complete data for BASDAI-fatigue, and only 167 had available data for SF-36 and SF-36 VT. The baseline characteristics of the patients are detailed in Table 1. 207 (79%) were men and 216 (82.4%) were of Chinese ethnicity. The mean (standard deviation, SD) age was 41.7 (13.7) years, while that of duration of disease was 10.1 (8.3) years (Table 1).

**Table 1:** Baseline characteristics of patients with axSpA.

**Characteristics**	**Total axSpA (*n* = 262)**	**BASDAI-F criteria**		**SF-36 VT criteria**	
		**No severe fatigue (*n* = 117)**	**Severe fatigue (*n* = 145)**	**No severe fatigue (*n* = 115)**	**Severe fatigue (*n* = 52)**
**Social demographics**					
Age, years	41.7 (13.7)	42.4 (15.1)	41.2 (12.5)	41.0 (13.2)	38.6 (11.9)
Men, *n* (%)	207 (79)	97 (82.9)	110 (75.9)	96 (83.5)	37 (71.2)
Chinese, *n* (%)	216 (82.4)	92 (78.6)	124 (85.5)	94 (81.7)	43 (82.7)
Secondary education and below, *n* (%)	52 (26.8)	14 (16.7)	38 (34.5)^**^	21 (21.6)	13 (31.0)
Education (1–7), median (IQR)	6.0 (5.0–7.0)	6.0 (6.0–7.0)	6.0 (5.0–7.0)^*^	6.0 (6.0–7.0)	6.0 (5.0–7.0)
Public housing, *n* (%)	150 (78.5)	57 (69.5)	93 (85.3)^**^	74 (77.9)	35 (83.3)
Housing type (1–7), median (IQR)	4.0 (3.0–5.0)	4.0 (3.0–6.0)	3.0 (3.0–4.0)	4.0 (3.0–5.0)	3.0 (3.0–5.0)
BMI, kg/m^2^	24.6 (6.1)	23.9 (6.1)	25.2 (6.0)^*^	24.4 (5.4)	24.9 (7.0)
**Clinical characteristics**					
rAxSpA, *n* (%)	215 (82.1)	101 (86.3)	114 (78.6)	91 (79.1)	45 (86.5)
Duration of disease, years	10.1 (8.3)	10.4 (9.1)	9.9 (7.7)	9.4 (8.7)	9.1 (7.4)
BASMI_10_ (0–10)	3.4 (1.8)	3.2 (1.8)	3.5 (1.8)	3.2 (1.8)	3.6 (1.7)
Tender joint count (0–68)	0.3 (1.8)	0.2 (2.0)	0.4 (1.5)^*^	0.1 (0.4)	0.8 (2.4)
Swollen joint count (0–66)	0.2 (1.1)	0.1 (0.3)	0.3 (1.5)	0.1 (0.4)	0.7 (2.3)
Clinically damaged joints (0–68)	0.1 (0.8)	0.2 (1.0)	0.1 (0.5)	0.0 (0.0)	0.1 (0.5)^**^
SPARCC enthesitis index (0–16)	0.4 (1.5)	0.2 (1.0)	0.6 (1.8)^*^	0.2 (1.1)	0.7 (2.1)^*^
ESR, mm/h	24.3 (23.6)	24.2 (23.6)	24.4 (23.6)	23.6 (23.6)	27.4 (24.0)
**Treatment**					
Current NSAIDs, *n* (%)	211 (80.5)	94 (80.3)	117 (80.7)	91 (79.1)	43 (82.7)
Current steroids, *n* (%)	11 (4.2)	2 (1.7)	9 (6.2)	2 (1.7)	5 (9.6)^*^
Current DMARDs, *n* (%)	88 (33.6)	36 (30.8)	52 (35.9)	33 (28.7)	18 (34.6)
Current biologics, *n* (%)	36 (13.7)	13 (11.1)	23 (15.9)	14 (12.2)	10 (19.2)
**Patient reported outcomes**					
BASDAI (0–10)	3.6 (1.9)	2.4 (1.3)	4.7 (1.7)^***^	3.1 (1.6)	5.0 (1.8)^***^
BASDAI-axial pain (0–10)	4.4 (2.5)	3.1 (2.2)	5.5 (2.3)^***^	4.1 (2.5)	5.7 (2.2)^***^
BASDAI-peripheral pain (0–10)	2.5 (2.8)	1.5 (1.8)	3.3 (3.1)^***^	1.8 (2.2)	4.5 (3.0)^***^
BASDAI-morning stiffness (0–10)	3.7 (2.5)	3.0 (2.3)	4.2 (2.5)^***^	3.3 (2.3)	4.9 (2.6)^***^
BAS-G (0–100)	40.1 (20.2)	31.9 (18.2)	46.8 (19.3)^***^	39.2 (17.3)	52.1 (19.0)^***^
BASFI (0–10)	2.3 (2.1)	1.4 (1.6)	3.0 (2.3)^***^	1.8 (2.0)	3.4 (2.2)^***^
ASQoL (0–18)	3.7 (4.2)	2.3 (3.2)	4.9 (4.6)^***^	3.2 (3.6)	8.2 (3.9)^***^
**SF-36 Domains^¥^**					
PF (0–100)	73.7 (22.3)	83.1 (18.5)	66.7 (22.5)^***^	79.6 (19.7)	60.5 (22.5)^***^
RP (0–100)	73.5 (24.7)	81.1 (23.1)	67.9 (24.5)^***^	79.3 (22.6)	60.7 (24.7)^***^
BP (0–100)	57.0 (21.4)	66.4 (17.6)	50.2 (21.5)^***^	64.6 (19.0)	40.4 (16.8)^***^
GH (0–100)	53.7 (20.6)	58.1 (21.0)	50.5 (19.9)^*^	59.4 (18.8)	41.2 (18.9)^***^
VT (0–100)	57.4 (18.2)	65.9 (18.1)	51.1 (15.7)^***^	66.7 (13.2)	36.8 (7.9)^***^
SF (0–100)	75.8 (22.1)	85.6 (16.9)	68.6 (22.9)^***^	82.8 (18.2)	60.3 (22.2)^***^
RE (0–100)	79.4 (23.0)	84.9 (21.5)	75.4 (23.3)^**^	85.2 (19.7)	66.7 (24.6)^***^
MH (0–100)	69.9 (19.1)	75.4 (18.1)	65.8 (18.9)^**^	76.7 (15.4)	54.8 (17.8)^***^
PCS NBS	41.7 (12.0)	46.1 (10.0)	38.5 (12.4)^***^	44.6 (10.4)	35.5 (13.1)^***^
MCS NBS	43.8 (11.3)	47.4 (11.4)	41.1 (10.6)^***^	48.6 (9.1)	33.2 (8.2)^***^

*All figures are given in mean (SD) unless otherwise specified*.

*BMI, body mass index; rAxSpA, radiographic axial spondyloarthritis; BASMI_10_, Bath Ankylosing Spondylitis Metrology Index; SPARCC, Spondyloarthritis Research Consortium of Canada; ESR, erythrocyte sedimentation rate; NSAIDs, non-steroidal anti-inflammatory drugs; DMARDs, disease-modifying anti-rheumatic drugs; BASDAI, Bath Ankylosing Spondylitis Disease Activity Index; BAS-G, Bath Ankylosing Spondylitis Global Score; BASFI, Bath Ankylosing Spondylitis Functional Index; ASQoL, Ankylosing Spondylitis Quality of Life; SF-36, Short Form-36; PF, physical function; RP, role-physical; BP, bodily pain; GH, general health; VT, vitality; SF, social functioning; RE, role-emotional; MH, mental health; PCS, physical component summary; MCS, mental component summary; NBS, norm-based scoring*.

¥ *Data available in 167 patients who completed the SF-36*;

* *p < 0.05*;

** *p < 0.01*;

*** *p < 0.001*.

Among the 262 patients, 145 (55.3%) reported severe fatigue by the BASDAI-fatigue criteria (BASDAI-fatigue ≥ 5/10). Among the 167 patients with data for SF-36, 52 (31.1%) had severe fatigue by the SF-36 VT criteria (VT ≤ 47.6/100). Patients who had severe fatigue by the BASDAI-fatigue and SF-36 VT criteria had worse scores across all disease-activity assessments (BASDAI, axial pain, peripheral joint pain, morning stiffness, SPARCC enthesitis index) as well as PROs measuring disease impact (BAS-G, BASFI, ASQoL, and all SF-36 domains), compared to those without severe fatigue. Additionally, patients with severe fatigue by the BASDAI-fatigue criteria had higher BMI and lower education levels, with a higher proportion of them reporting an education level of secondary or below and living in public housing, compared to those without severe fatigue. Patients who had severe fatigue by the SF-36 VT criteria had a higher damaged joint count compared to those without severe fatigue.

**Figure 1:**
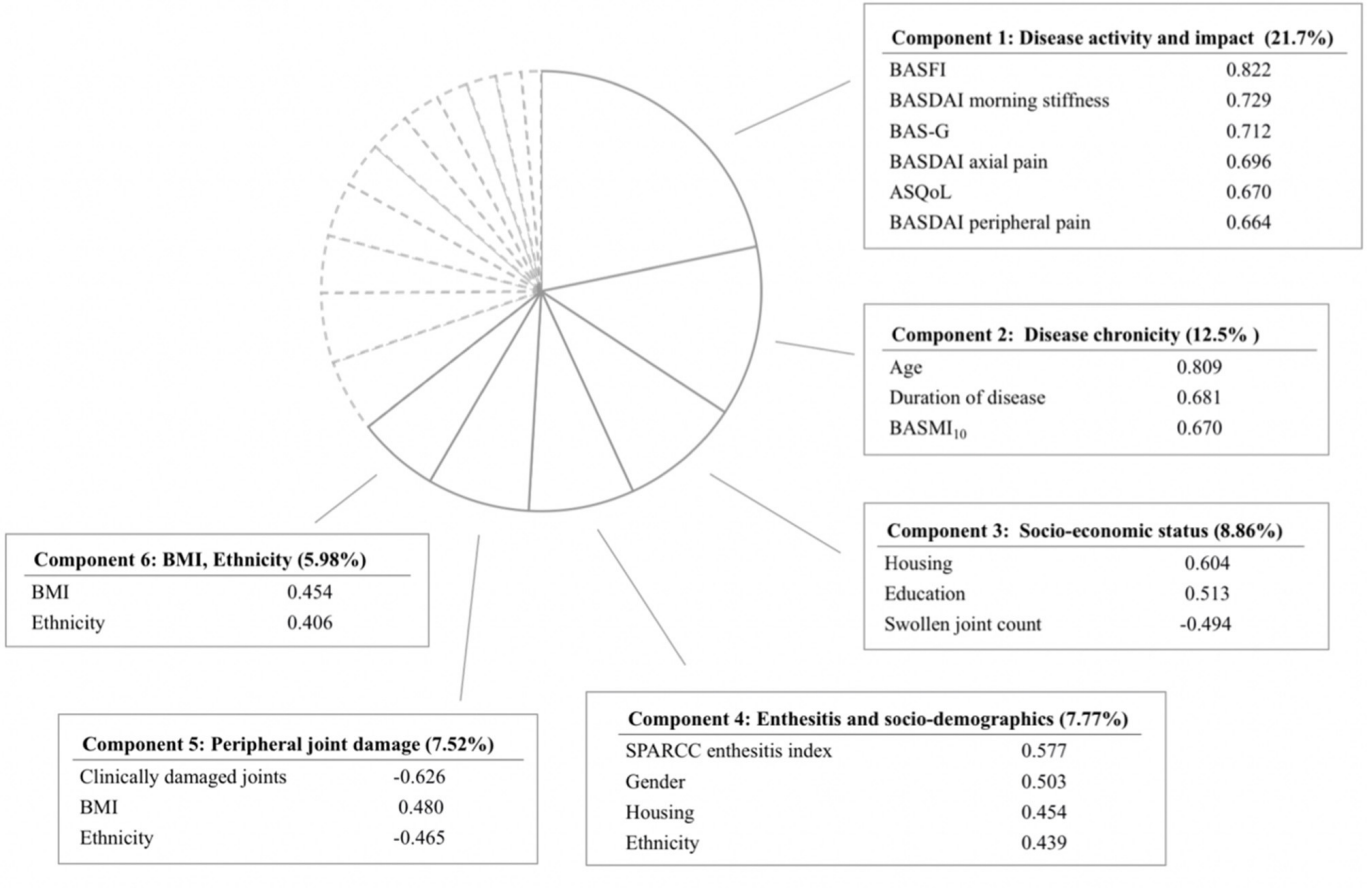
Principal component analysis yielding 6 components with eigenvalue greater than 1. Only variables with high factor loading are shown. BASFI, Bath Ankylosing Spondylitis Functional Index; BASDAI, Bath Ankylosing Spondylitis Disease Activity Index; BAS-G, Bath Ankylosing Spondylitis Global Score; ASQoL, Ankylosing Spondylitis Quality of Life; BASMI10, Bath Ankylosing Spondylitis Metrology Index; SPARCC, Spondyloarthritis Research Consortium of Canada; BMI, Body Mass Index.

In the PCA, we included gender, ethnicity, age, education, housing type, body mass index (BMI), duration of disease, swollen joint count, SPARCC enthesitis index, BASMI_10_, clinically damaged joints, BASDAI-axial pain, BASDAI-peripheral joint pain, BASDAI-morning stiffness, BAS-G, BASFI, and ASQoL. BASDAI-axial pain and BASDAI-peripheral joint pain were included separately as they represent distinctive domains of disease activity. Tender joint count was excluded due to collinearity with swollen joint count. Due to a sizable amount of missing data for SF-36, we chose ASQoL over the SF-36 domains into the model. Six principal components explaining 64.4% of the total variance (Figure 1 and Supplementary Table 1) were derived. From components 1 to 6, the concepts represented are disease activity and impact, disease chronicity, socio-economic status, enthesitis and socio-demographics, peripheral joint damage, and BMI/ethnicity, respectively. Component 1, explaining 21.7% of the variance, reflected disease activity, and impact. The variables included are BASFI, BASDAI-morning stiffness, BAS-G, BASDAI-axial pain, ASQoL, and BASDAI-peripheral joint pain. Component 2, accounting for 12.5% of the variance, represented the concept of disease chronicity and comprised of age, duration of disease and BASMI_10_. In the regression model analysis, only component 1 was significantly associated with BASDAI-fatigue (*p* < 0.001), with the model accounting for 30.1% of variance in BASDAI-fatigue. In the SF-36 VT model, both components 1 and 2 were significantly associated with fatigue (*p* < 0.001), with the model explaining 44.2% of variance in VT.

In the univariable analysis (Table 2), BASDAI-fatigue correlated strongly with BASDAI-axial pain; moderately with BAS-G, BASFI, BASDAI-morning stiffness, and ASQoL; and weakly with BASDAI-peripheral joint pain, SPARCC enthesitis index and education. In contrast, SF-36 VT correlated strongly with ASQoL; moderately with BASFI, BAS-G, BASDAI-peripheral joint pain, BASDAI-morning stiffness, and BASDAI-axial pain; and weakly with clinically damaged joints, SPARCC enthesitis index and age.

**Table 2:** Spearman's rank correlation between fatigue measures and study variables.

	**BASDAI-F**		**SF-36 VT**	
	**Correlation coefficient**	***p*-value**	**Correlation coefficient**	***p*-value**
Age	−0.069	0.269	0.162	0.037
Education	−0.158	0.028	0.116	0.174
Housing	−0.083	0.254	0.114	0.185
BMI	0.100	0.115	0.069	0.385
Duration of disease	0.031	0.617	0.005	0.948
BASMI_10_	0.070	0.256	−0.079	0.311
Swollen joint count	0.108	0.081	−0.135	0.083
Clinically damaged joints	−0.028	0.648	−0.200	0.010
SPARCC enthesitis index	0.196	0.001	−0.178	0.021
ESR	0.046	0.495	−0.059	0.479
BASDAI-axial pain	0.529	<0.001	−0.346	<0.001
BASDAI-peripheral pain	0.289	<0.001	−0.414	<0.001
BASDAI-morning stiffness	0.347	<0.001	−0.376	<0.001
BAS-G	0.468	<0.001	−0.424	<0.001
BASFI	0.427	<0.001	−0.449	<0.001
ASQoL	0.335	<0.001	−0.634	<0.001

*BMI, body mass index; BASMI_10_, Bath Ankylosing Spondylitis Metrology Index; SPARCC, Spondyloarthritis Research Consortium of Canada; ESR, erythrocyte sedimentation rate; BASDAI, Bath Ankylosing Spondylitis Disease Activity Index; BAS-G, Bath Ankylosing Spondylitis Global Score; BASFI, Bath Ankylosing Spondylitis Functional Index; ASQoL, Ankylosing Spondylitis Quality of Life*.

In the multivariable analysis, we selected variables with *p* < 0.2 in the univariable analysis, which included education, swollen joint count, SPARCC enthesitis index, BASDAI-axial pain, BASDAI-peripheral joint pain, BASDAI-morning stiffness, BAS-G, BASFI, and ASQoL. We also included gender (5, 8), age (14), and BMI (41) as these were previously reported associations with fatigue. Ethnicity and housing type as a surrogate of socio-economic status (27) were included for theoretical exploration. In addition, we included duration of disease, clinically damaged joints and BASMI_10_ to explore possible associations between fatigue and the concepts of disease chronicity and damage (peripheral and axial). The results of the multivariate analysis are presented in Table 3. In the BASDAI-fatigue model, BASDAI-axial pain, BASFI, BAS-G, and ethnicity were significantly associated with fatigue. In the SF-36 VT model, ASQoL, and BASDAI-morning stiffness were significantly associated with fatigue.

**Table 3:** Factors associated with BASDAI-F and SF-36 VT on multiple linear regression.

**Variables**	***b***	**95% CI**	***p*-value**
**BASDAI-F**			
BASDAI-axial pain	0.214	0.079, 0.348	0.002
BASFI	0.238	0.077, 0.399	0.004
BAS-G	0.024	0.006, 0.041	0.009
Ethnicity	0.894	0.119, 1.669	0.024
**SF-36 VT**			
ASQoL	−2.346	−2.931, −1.760	<0.001
BASDAI-morning stiffness	−1.368	−2.348, −0.388	0.007

*Variables entered into both models: gender, ethnicity (Chinese/Non-Chinese), age, education (1–7), housing type (1–7), BMI, duration of disease, BASMI_10_, SPARCC enthesitis index, swollen joint count, clinically damaged joints, BASDAI-axial pain, BASDAI-peripheral pain, BASDAI-morning stiffness, BAS-G, BASFI, and ASQoL*.

*BASDAI, Bath Ankylosing Spondylitis Disease Activity Index; BASFI, Bath Ankylosing Spondylitis Functional Index; BAS-G, Bath Ankylosing Spondylitis Global Score; ASQoL, Ankylosing Spondylitis Quality of Life*.

## Discussion

Fatigue is prevalent in our study cohort, underscoring its importance as a major symptom in axSpA. In our study, 55.3% of all patients experienced severe fatigue by the BASDAI-fatigue criteria (BASDAI-fatigue ≥ 5/10) while 31.1% experienced severe fatigue by the SF-36 VT criteria (VT ≤ 10 percentile of the general population). Patients who experienced severe fatigue generally reported higher disease activity and greater perceived disease impact. In addition, patients with severe fatigue by the BASDAI-fatigue criteria had higher BMI and lower socio-economic status. In the PCA, disease activity and impact variables were associated with both measures of fatigue, and disease chronicity was additionally associated with SF-36 VT. In the multivariable analyses, BASDAI-axial pain, BASFI, BAS-G, and ethnicity (non-Chinese vs. Chinese) were associated with BASDAI-fatigue; while ASQoL and BASDAI-morning stiffness were associated with SF-36 VT.

The prevalence of severe fatigue in our axSpA cohort was largely concordant with that reported in previous studies. In several Western cohorts using the same definition (BASDAI-fatigue ≥ 5/10), the prevalence of fatigue ranged from 49 to 66.4% (5–10). In a Norwegian study, which defined severe fatigue as SF-36 VT ≤ 10th percentile of the general population, 32% of their patients with axSpA were found to have severe fatigue (8). In our study, disease activity assessments stood out consistently as key predictors of fatigue. Disease activity is a well-recognized driver of fatigue in axSpA (5–10, 14, 15, 17). This suggests a component of fatigue that is amenable to disease modifying treatment. In fact, studies have demonstrated that biologics therapy reduced fatigue levels, albeit suboptimally (6), highlighting the multi-factorial nature of fatigue in axSpA. ESR, which may not be a good reflection of disease activity (43), was not associated with fatigue in our study. This is consistent with findings from other studies (8, 17). C-reactive protein may be a better reflection of disease activity but was not consistently captured in our study. Besides disease activity, we also found significant associations between disease impact and fatigue. In the multi-variable analysis, physical function (measured by BASFI), and BAS-G were associated with BASDAI-fatigue, while ASQoL was associated with SF-36 VT. Our findings are consistent with other studies that reported BASFI (7–9, 14, 15, 17), BAS-G (5, 9, 14), and ASQoL (6, 9, 14, 15) as predictors of fatigue. This highlights patients' perception of fatigue as a disabling symptom and accentuates the importance of recognizing and addressing fatigue in clinical practice. Moreover, poor mental health including anxiety and depression has been found to be associated with fatigue (7, 8, 10, 14, 15, 32). In a study on patients with ankylosing spondylitis using brain magnetic resonance imaging, the left thalamus volume of patients with severe fatigue was significantly larger than that of healthy controls and patients without fatigue (32), suggesting a role of the central nervous system in the subjective symptoms of fatigue. This emphasizes the need for a holistic bio-psycho-cognitive approach in the clinical management of fatigue in axSpA. In our study, we also found an inverse correlation between damaged joint count and SF-36 VT. We postulate that joint damage could possibly increase the effort required in performing daily activities, resulting in greater fatigue, as hypothesized in studies in rheumatoid arthritis (44), where peripheral joint damage features prominently. Spinal damage, which was measured by BASMI_10_ and clustered together with age and disease duration in the PCA, was associated with SF-36 VT. However, it was not associated with fatigue measures in the multivariable analysis, which is in keeping with other studies (6–9, 15, 17).

Several patient-related factors were associated with fatigue in our study. We found that Chinese ethnicity is significantly associated with BASDAI-fatigue in the multivariable analysis after adjusting for other variables. Some recent studies have found that Blacks, African Americans, and African Brazilians have higher disease activity compared to Whites (19–21), suggesting that ethnicity may influence disease activity in axSpA. We also found that patients with severe BASDAI-fatigue had higher BMI, though statistical significance was lost in the multivariable analyses. This is consistent with other studies (17, 41), and could possibly be explained by a heightened level of pro-inflammatory cytokines resulting from increased adiposity (45), which is increasingly recognized for its immunological properties. Obesity has been associated with various disease outcomes in SpA (24, 46). Clinicians should therefore consider weight loss in the management of fatigue in patients with high BMI. Moreover, we found that patients with severe BASDAI-fatigue had lower socio-economic status, as measured by education and housing type. Housing type is a unique surrogate of socio-economic status in Singapore (47). Socio-economic status is a well-known determinant of health (48), and may influence disease activity as well as the progression of spinal damage in axSpA (49, 50). Lower education levels were similarly reported to be associated with higher fatigue levels in a French cohort (5). Age positively correlated with SF-36 VT in our study, but not with BASDAI-fatigue. This could possibly be due to the greater discrepancy in vitality perceived by younger patients when they compare themselves to their healthy peers. Except for a Dutch study (14), previous studies have largely reported no association between age and fatigue (15, 17). Some studies have demonstrated that women have higher fatigue levels compared to men (5, 8). In our study, gender was not significantly associated with fatigue. This could possibly be ascribed to the small sample size of women in our cohort.

Our study is one of the few (17) to evaluate factors associated with axSpA-fatigue in an Asian cohort, thereby supplementing current limited understanding on fatigue amongst patients with axSpA in Asia. Moreover, our preliminary data allowed for the study of fatigue among different Asian ethnicities. However, ethnicity was eventually analyzed as a dichotomous variable (Chinese vs. non-Chinese) due to limited representation from other ethnicities, thus limiting generalizability of the finding to other Asian ethnicities. We assessed fatigue using two different measures, which allowed for comparison and identification of variables consistently associated with both measures of fatigue. However, we recognize that our study has several limitations. Firstly, the sample size of our cohort is small. Secondly, this is a cross-sectional study and hence it is not possible to establish the causal relationship between fatigue and the variables. In addition, a third of our patients did not have data captured for SF-36. This resulted from a non-differential omission in distributing the SF-36 questionnaires on some clinic days where research assistant support was not available. However, the baseline characteristics of patients who completed the SF-36 were not different from those who did not. Moreover, a sensitivity analyses for BASDAI-fatigue limited to the 167 patients with SF-36 data yielded consistent results (data not shown). Lastly, variables which could potentially be linked to fatigue such as work productivity, sleep disturbance, anxiety, depression, fibromyalgia, and previous use of medication including traditional Chinese medicine were not evaluated in this study. These could have accounted for some of the unexplained variance in the PCA.

In conclusion, fatigue is prevalent amongst patients with axSpA in Singapore. Disease activity is a key predictor of fatigue in our study, suggesting that disease modifying treatment should continue to be a cardinal pillar in the management of fatigue. We also found that fatigue has significant associations with patients' perception of well-being, physical function, and quality of life. In addition, patient-related factors such as ethnicity, BMI, and socio-economic status were linked to fatigue. This study underscores the multi-factorial nature of fatigue amongst Asian patients with axSpA and emphasizes the need for a holistic approach in the management of fatigue.

## Data Availability Statement

The data analyzed in this study is subject to the following licenses/restrictions: All data are available upon reasonable request to the corresponding author. Requests to access these datasets should be directed to katy.leung.y.y@singhealth.com.sg.

## Ethics Statement

The studies involving human participants were reviewed and approved by SingHealth Centralized Institutional Review Board. The patients/participants provided their written informed consent to participate in this study.

## Author Contributions

YYL and WF conceptualized the study design and performed the data collection. WZL and YYL performed the statistical analysis. All authors authored and reviewed the manuscript and approved the version of this article to be published.

## Conflict of Interest

The authors declare that the research was conducted in the absence of any commercial or financial relationships that could be construed as a potential conflict of interest.
