# Supplementary material for: Exploring the Prevalence and Factors Associated With Fatigue in Axial Spondyloarthritis in an Asian Cohort in Singapore
Source: Front Med (Lausanne). 2021 Feb 4;8:603941. doi: 10.3389/fmed.2021.603941 (PMC7890247; doi:10.3389/fmed.2021.603941)
Supplement: Supplementary file 1 [file Table_1.DOCX]

**Supplementary Table 1.** Summary of principle component analysis

|  | Factor loading onto components | | | | | |
| --- | --- | --- | --- | --- | --- | --- |
|  | **1** | **2** | **3** | **4** | **5** | **6** |
| BASFI (0-10) | **0.822** | 0.054 | 0.020 | -0.229 | -0.046 | -0.154 |
| BASDAI-morning stiffness (0-10) | **0.729** | -0.080 | 0.301 | 0.086 | -0.073 | 0.031 |
| BAS-G (0-100) | **0.712** | -0.127 | 0.241 | 0.021 | 0.235 | 0.004 |
| BASDAI-axial pain (0-10) | **0.696** | -0.159 | 0.281 | -0.111 | 0.013 | -0.029 |
| ASQoL (0-18) | **0.670** | -0.407 | -0.180 | 0.072 | 0.076 | 0.119 |
| BASDAI-peripheral pain (0-10) | **0.664** | -0.133 | -0.043 | 0.205 | -0.120 | 0.180 |
| Age | 0.112 | **0.809** | 0.009 | 0.055 | 0.159 | -0.250 |
| Duration of disease | 0.179 | **0.681** | 0.346 | 0.234 | -0.018 | 0.111 |
| BASMI_10_ (0-10) | 0.443 | **0.670** | -0.024 | -0.206 | 0.005 | -0.225 |
| Housing (1-7) | -0.135 | 0.010 | **0.604** | **0.454** | 0.045 | 0.236 |
| Education (1-7) | -0.289 | -0.271 | **0.513** | 0.248 | 0.272 | -0.197 |
| Swollen joint count (0-66) | 0.395 | -0.095 | **-0.494** | 0.166 | 0.402 | 0.209 |
| SPARCC enthesitis index (0-16) | 0.175 | -0.104 | -0.142 | **0.577** | -0.251 | -0.401 |
| Gender | 0.115 | 0.042 | -0.403 | **0.503** | 0.173 | -0.376 |
| Clinically damaged joints (0-68) | 0.246 | 0.109 | -0.028 | -0.154 | **-0.626** | 0.089 |
| BMI | 0.046 | 0.397 | -0.154 | 0.039 | **0.480** | **0.454** |
| Ethnicity | -0.019 | 0.258 | -0.212 | **0.439** | **-0.465** | **0.406** |
| Cumulative variance explained (%) | 21.7 | 34.3 | 43.2 | 50.9 | 58.4 | 64.4 |

Abbreviations: BASFI, Bath Ankylosing Spondylitis Functional Index; BASDAI, Bath Ankylosing Spondylitis Disease Activity Index; BAS-G, Bath Ankylosing Spondylitis Global Score; ASQoL, Ankylosing Spondylitis Quality of Life; BASMI_10_, Bath Ankylosing Spondylitis Metrology Index; SPARCC, Spondyloarthritis Research Consortium of Canada; BMI, body mass index
